# A Pilot Study Exploiting the Industrialization Potential of Solid Lipid Nanoparticle-Based Metered-Dose Inhalers

**DOI:** 10.3390/pharmaceutics15030866

**Published:** 2023-03-07

**Authors:** Lei Shu, Wenhua Wang, Chon-iong Ng, Xuejuan Zhang, Ying Huang, Chuanbin Wu, Xin Pan, Zhengwei Huang

**Affiliations:** 1 College of Pharmacy, Jinan University, Guangzhou 510006, China; 2 School of Pharmaceutical Sciences, Sun Yat-sen University, Guangzhou 510275, China

**Keywords:** inhalation, solid lipid nanoparticle, metered-dose inhaler, pilot study, nanocoating

## Abstract

Background: Delivery of inhalable nanoparticles through metered-dose inhalers (MDI) is a promising approach to treat lung disease such as asthma and chronic obstructive pulmonary disease. Nanocoating of the inhalable nanoparticles helps in stability and cellular uptake enhancement but complicates the production process. Thus, it is meaningful to accelerate the translation process of MDI encapsulating inhalable nanoparticles with nanocoating structure. Methods: In this study, solid lipid nanoparticles (SLN) are selected as a model inhalable nanoparticle system. An established reverse microemulsion strategy was utilized to explore the industrialization potential of SLN-based MDI. Three categories of nanocoating with the functions of stabilization (by Poloxamer 188, encoded as SLN(0)), cellular uptake enhancement (by cetyltrimethylammonium bromide, encoded as SLN(+)), and targetability (by hyaluronic acid, encoded as SLN(−)) were constructed upon SLN, whose particle size distribution and zeta-potential were characterized. Subsequently, SLN were loaded into MDI, and evaluated for the processing reliability, physicochemical nature, formulation stability, and biocompatibility. Results: The results elucidated that three types of SLN-based MDI were successfully fabricated with good reproducibility and stability. Regarding safety, SLN(0) and SLN(−) showed negligible cytotoxicity on cellular level. Conclusions: This work serves as a pilot study for the scale-up of SLN-based MDI, and could be useful for the future development of inhalable nanoparticles.

## 1. Introduction

Inhalable nanoparticles have been widely reported in the literature to treat lung diseases such as lung carcinoma [1], systemic diseases such as diabetes [2], and to act as vaccine carriers [3]. A literature survey via PubMed suggested that 2760 relevant papers were published since 1998 (searching date: 11 January 2023). Their pharmacodynamic effects are demonstrated in animal models and have shown good therapeutic efficacies in patients. Clinical translation of inhalable nanoparticles may provide promising solutions to serious chronic illness such as respiratory diseases.

It should be noted that the prerequisite for the clinical pulmonary delivery of inhalable nanoparticles is a satisfactory deposition outcome. According to the previous study, transforming nanoparticles into micro-sized aerosols with appropriate aerodynamic attributes can achieve satisfactory deposition [4]. As inhalable nanoparticles are generally fabricated in liquid state, encapsulating into nebulizers and metered-dose inhalers (MDI) that can transform individual nanoparticles into micro-sized aerosols with proper aerodynamic characteristics is a promising settlement. Compared to nebulizers, MDI are portable and easy to use [5]. Hence, developing a nanoparticles-containing MDI is of great significance.

However, there are no inhalable nanoparticles in MDI forms currently available in the market. The nanocoating structure of inhalable nanoparticles is a major obstacle for its industrialization. The term nanocoating refers to the surface modification layer on the nanoparticles for the stabilization [6], cellular uptake enhancement [7], and to achieve the targetability [8]. A range of synthetic polymers such as poloxamer, [poly(ethylene oxide)-poly(propylene oxide)-poly(ethylene oxide) block copolymer [9], surfactants such as Tween [10] and cetyltrimethylammonium bromide (CTAB) [11], and natural substances such as chitosan [12], hyaluronic acid (HA) [13], and folate [14] have been used for nanocoating. The loading of inhalable nanoparticles into MDI includes a high-pressure propellant filling process [15], which may compromise the nanocoating functions. A multilayer nanocoating with different materials further complicate the design and development process. Hence, although the nanocoating endows inhalable nanoparticles with aforementioned properties, the difficulty in industrialization for inhalable nanoparticles-based MDI is substantially increased. It is urgent to exploit new strategies to facilitate the industrialization of related products.

A reverse microemulsion technology [16] to produce nanoparticles-incorporated MDI has been reported by our group [17]. The emulsification layer of reverse microemulsion system consist of surfactant and co-surfactant that can sterically refrain the nanocoating and protect it from collapse during MDI filling process. By exploiting the applicability of this strategy on a larger scale, the industrialization progress of inhalable nanoparticles-based MDI may be triggered.

**Figure 1:**
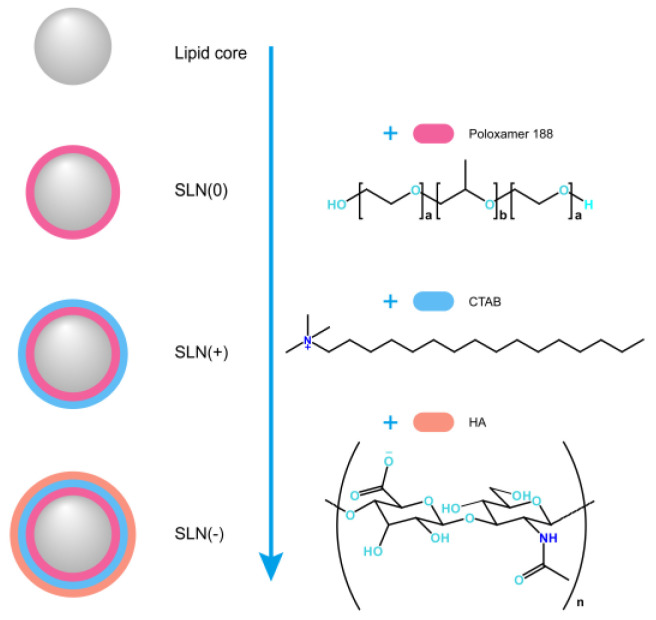
Scheme for the involved nanoparticles. Abbreviations in the figure: SLN- solid lipid nanoparticles; CTAB- cetyltrimethylammonium bromide; HA- hyaluronic acid.

In this study, the industrialization potential of MDI loading nanoparticles with different nanocoating was preliminarily investigated. A multilayer nanocoating was introduced to scrutinize the robustness of production. A typical lipid-based nanomedicine, solid lipid nanoparticles (SLN) was selected as the model inhalable nanoparticles and coated rationally with Poloxamer 188 (representing the stabilization function), Poloxamer 188 plus CTAB (positive surface charge, representing the cellular uptake enhancement function), and Poloxamer 188 plus CTAB plus HA (negative surface charge, representing the targetability function). The involved nanoparticles and the nanoarchitecture thereof were summarized in Figure 1. The nanostructure design was partly referred to the literature [13].

## 2. Materials and Methods

### 2.1. Materials

Palmityl palmitate and absolute ethanol were purchased from Aladdin Industrial, Inc. (Shanghai, China). Poloxamer 188 was supplied by BASF SE (Ludwigshafen, Germany). CTAB was obtained from Sinopharm Chemical Reagent Co., Ltd. (Shanghai, China). HA (average molecular weight: 1 MDa) and dimethyl sulfoxide (DMSO) were purchased from Macklin Inc. (Shanghai, China). Pluronic L64 was supplied by Wengjiang Chemical Inc. (Shaoguan, China). Methylthiazolyldiphenyl-tetrazolium bromide (MTT) was obtained from Merck KGaA (Darmstadt, Germany). Phosphate buffer saline (PBS), DMEM media, fetal bovine serum, and trypsin were purchased from Thermo Fisher Scientific Inc. (Waltham, MA, USA). Propellant HFA 134a (chemically 1, 1, 1, 2-tetrafluoroethane) was supplied by Kemu Chemical Inc. (Shanghai, China). A549 cells (ATCC code: CCL-185) were purchased from National Collection of Authenticated Cell Cultures (Shanghai, China). Ultra-pure water provided by PureLAB Option system (ELGA Lab Water, Inc., High Wycombe, UK) was used throughout this study.

### 2.2. SLN Preparation

SLN were prepared according to the documented method [13]. Palmityl palmitate (150 mg) and Poloxamer 188 (25 mg) were placed in a vessel and melted at 80 °C. Then, 5 mL of water was added dropwise into the melted mixture and the system was stirred for 5 min at an oscillator. Ultrasonication (BILON-650Y, BiLon Instrument Co., Ltd., Shanghai, China) in an ice bath was applied with a 20% power and 2 s/2 s working/rest cycling. This formulation was encoded as SLN(0) (undecorated SLN).

CTAB solution with a concentration of 3.75 mg·mL^−1^ was prepared by dissolving 3.75 g CTAB solid in 1 L water. Then, 100 μL of CTAB solution was diluted by 5 mL water. The rest procedures were as described above and this formulation was named as SLN(+) (positive charge decorated SLN). SLN(+) was mixed with HA solution (0.625 mg·mL^−1^) at a volume ratio of 5:4, and the system was stirred at ambient temperature for 30 min to produce SLN(−) (negative charge decorated SLN).

### 2.3. Particle Size Distribution and Zeta-Potential Characterization

#### 2.3.1. Particle Size

SLN(0), SLN(+), and SLN(−) were diluted 120 times and 1 mL of the samples was equilibrated for 2 min and subjected to particle size measurement via Nano ZS90 (Malvern Instruments Ltd., Malvern, UK). Average size and polydispersity index (PDI) were measured using Nano ZS90 and were referred to prior to data analysis.

#### 2.3.2. Autocorrelation Function Curve

The change of autocorrelation function along with time was recorded during particle size determination, within 10^8^ µs. The function value was normalized by setting the initial value as 1, and an average of three functions was plotted as a curve. The quality reports by Nano ZS90 were referred to prior to data analysis.

#### 2.3.3. Zeta-Potential

The samples in Section 2.3.1 were also subjected to zeta-potential determination, through the corresponding module of Nano ZS90. Each sample was determined in triplicates. The quality reports by Nano ZS90 were referred to prior to data analysis.

### 2.4. MDI Filling

Before formal filling procedure, the pipeline of type 15 filling machine (Zhihua Aerosol Equipment Ltd., Zhongshan, China) was washed by HFA 134a and the vacuum pump of the filling machine was allowed for equilibrium.

The filling process of MDI had been reported elsewhere [18], and the content of components could be referred to the literature [17]. Pluronic L64, ethanol, and SLN (SLN(0), SLN(+) and SLN(−)) were added successively into the aerosol container, which was sealed by a 50-μL metered-dose valve. It was noticed that the fresh prepared SLN systems were used. The mixture was vortexed for 1 min, and HFA 134a were filled through the filling machine. The MDI products were allowed to stand for 10 min to recover to room temperature. MDI was manufactured continuously for at least three batches. A batch consisted of 15 MDI products.

### 2.5. MDI Evaluations

#### 2.5.1. Appearance

A laser pen (Deli Group Co., Ltd., Shanghai, China) was used to lignite the MDI products at a distance of 5 cm, and the photographs representing Tyndall’s effect were taken.

#### 2.5.2. Filling Reproducibility

Using the method in Section 2.4, ten products were prepared. The aerosol container and the MDI products were accurately weighed and recorded as *W*_0_ and *W*’, respectively. The weight of filled HFA 134a (Δ*W*) was calculated as follows:

Δ*W* = *W*_0_ − *W*’ (1)

The relative standard deviation (RSD) values of Δ*W* were calculated to evaluate the filling reproducibility.

#### 2.5.3. Total Shots

The MDI products were fired into a 500 mL beaker containing approximately 50 mL of water, until the formulations were aerosolized completely. The interval between shots were 5 s. The number of total shots was examined.

#### 2.5.4. Valve Precision

After discarding the first three shots, the MDI products were fired according to Section 2.5.3. Before and after each shot, the MDI products were weighted. The reducing weight per press was recorded to evaluate the valve precision.

#### 2.5.5. Leak Detection

The MDI products were stored in dark condition at 20 ± 2 °C temperature and 60 ± 5% relative humidity, for 15 d. On day 0, 3, 5, 10, and 15, the weight of MDI products was recorded to detect the possible leakage.

#### 2.5.6. Re-Dispersity

On day 0, 3, 5, and 10 of storage under the conditions described in Section 2.5.5, the MDI products were carefully retrieved and shaken manually for 30 s. The dispersity and suspending stability were inspected visually.

#### 2.5.7. Stability of Particle Size

The MDI products were stored according to Section 2.5.5, and the evolution of average size and PDI within 10 d was determined to assess the stability. Alongside with the test, instability phenomena such as flocculation, sedimentation, and layering were monitored visually.

### 2.6. Cytotoxicity Studies

A549 cells were cultivated using DMEM media and fetal bovine serum. They were cultivated to a confluence of 90% and processed by trypsin. The cells were transferred to 96-well plates at a density of 1 × 10^4^ mL^−1^. SLN-based MDI products were aerosolized and the residues were reconstructed with culture media adding into A549 cells at concentrations of 0.50, 0.75, 1.00, 1.50, 2.00, and 3.00 μg·mL^−1^. After incubation for 24 h and 72 h, the media was discarded and replaced by MTT solution (5 mg·mL^−1^) before incubating it for another 4 h. MTT solution was replaced by DMSO and the optical density (OD) was determined at 570 nm (OD_570_) and 630 nm (OD_630_) using a plate reader (Synergy H1, BioTek Instruments, Inc., Winooski, VT). The cell viability was calculated using the following Equation (2):

Cell viability (%) = (OD_570,sample_ − OD_630,sample_)/(OD_570,blank_ − OD_630,blank_) × 100% (2)

### 2.7. Statistics

The acquired data were expressed as mean ± standard deviation, wherever applicable, and then processed by statistical analysis via *t*-test or one-way analysis of variance (ANOVA). *p* < 0.05 was considered as a significant difference.

## 3. Results

This study exploited the industrialization potential of MDI based on a series of SLN with increasing layers of nanocoating. The model SLN were evaluated and then SLN-based MDI were produced and characterized to ensure the quality and reproducibility of the prepared formulation. Cytotoxicity of SLN-based MDI was tested to preliminarily highlight the administration safety.

### 3.1. Evaluations of SLN

**Figure 2:**
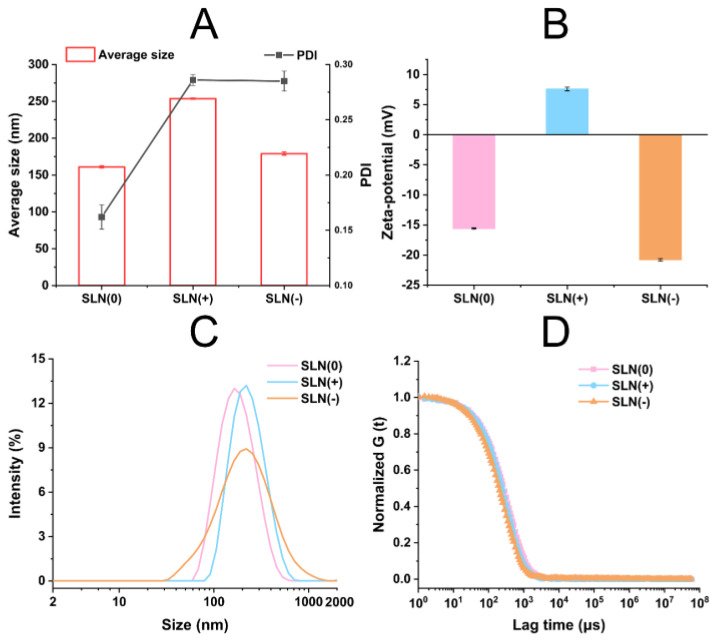
Characterizations. Average size and PDI (**A**), zeta-potential (**B**), particle size distribution curves (**C**), and autocorrelation function curves (**D**) (*n* = 3).

SLN(0) with nanocoating of Poloxamer 188, SLN(+) with nanocoating of Poloxamer 188 + CTAB, and SLN(−) with nanocoating of Poloxamer 188 + CTAB + HA were prepared through sonification method envisioning to achieve the stabilization, cellular uptake enhancement, and targetability, respectively. The particle size distribution and zeta-potential are shown in Figure 2.

The average size of SLN(0), SLN(+), and SLN(−) was measured to be ~160 nm, ~250 nm, and ~180 nm, respectively. And the PDI values were all recorded below 0.3, suggesting a relatively homogeneous nanoparticle distribution (Figure 2A). The low PDI values also indicated good storage stability. The average size of SLN(+) and SLN (−) was higher than that of SLN(0) (*p* < 0.05) due to the thickness of nanocoating. The markedly large size of SLN(+) was probably because of the large hydrolyzed diameter of CTAB nanocoating [19].

As shown in Figure 2B, SLN(+) and SLN(−) exhibited positive charge and negative charge, respectively, which was resulted from the natural charge of corresponding nanocoating. SLN(0) revealed a ~−15 mV zeta-potential might be due to adsorption of anions by Poloxamer 188 in the Stern layer [20]. SLN(−) possessed significantly higher negative charge than SLN(0) (*p* < 0.05), suggesting that the natural charge of HA was more intense than the absorbed anions.

The particle size distribution curve verified the homogeneity in Figure 2C and all the formulations were in monodisperse phase with only one peak. For the distribution curves, no frontal peak or tailed peak was found. The autocorrelation function curves representing the signal decay profile of all the formulations were similar (Figure 2D), indicating that the nanocoating did not induce the aggregation.

SLN with three types of nanocoating were successfully fabricated and characterized, viz., SLN(0), SLN(+), and SLN(−). The size distribution was homogeneous, which was favorable for subsequent scale-up studies.

### 3.2. Evaluations of SLN-Based MDI

SLN(0), SLN(+), and SLN(−) were produced into MDI through previously reported reverse microemulsion strategy. A series of characterizations was performed on the produced samples. Before reporting the results, it was worth mentioning that the lipid material of SLN, palmityl palmitate had negligible solubility in ethanol at ambient temperature (see Table S1 and the literature [21]) and therefore, addition of ethanol in the MDI filling process would not exert dissolution effect upon the SLN.

The photographs of MDI products displaying the Tyndall’s effect are provided in Figure S1. All the products were transparent with a pale gray color and a red laser beam was observed. The colloidal physicochemical nature of MDI products was demonstrated. No effect of the nanocoating was observed on the produce appearance.

**Figure 3:**
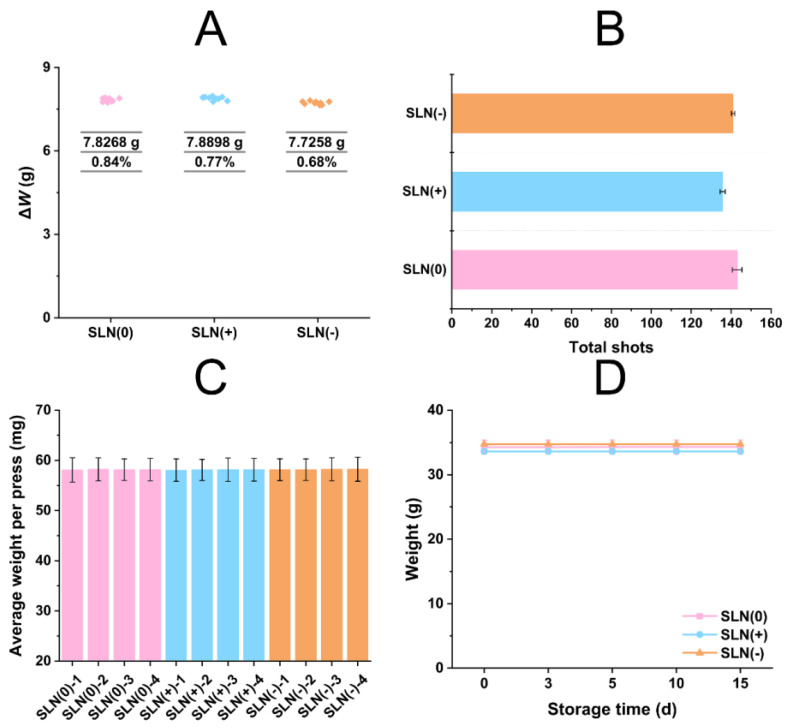
Evaluations of SLN-based MDI: Filling reproducibility (**A**), total shots (**B**), valve precision (**C**), and leak detection (**D**) (*n* = 10).

The propellant filling reproducibility was tested and the average filling amount of HFA 134a was measured ~7.8 g (Figure 3A) with no statistical difference (*p* > 0.05). Reproducibility was confirmed with low RSD (<1%). The numbers of total shots were around 140 (Figure 3B) suggesting that the products might be suitable for long-term usage. The total shots of SLN(+) was circa 135, lower than SLN(0) and SLN(−). The positive charge on SLN(+), might have contributed toward the physical interactions with glass container which led to enhanced stability of the formulation. The average weight per press was c.a. 58 mg with uniform and acceptable standard deviation values (Figure 3C). Valve precision was validated with low statistical difference (*p* > 0.05). The weight of MDI tested after 15 days of storage (*p* > 0.05) (Figure 3D) remained constant, elucidating no leakage. Additionally, during storage, after manual shaking, the MDI products could maintain stable dispersion for at least 5 min, facilitating the clinical administration. It was worth noticing that filling reproducibility and valve precision of all SLN systems had similar performance. We could infer that the performance of MDI filling process was reproducible in various formulations. Actually, filling reproducibility and valve precision were determined by the filling machine and had insignificant effect on the formulations.

**Figure 4:**
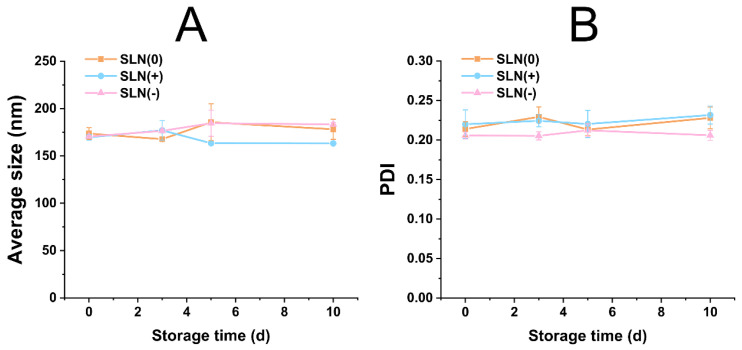
Evaluations of SLN-based MDI: evolution of average size (**A**) and PDI (**B**) versus time (*n* = 10).

The change in particle size distribution of MDI products was evaluated for 10 days (Figure 4). After encapsulation, the average size of different groups tuned to be approximately 175 nm (Figure 4A). The difference in the particle size of Figure 4A with Figure 2A might be due to the presence of Pluronic L64 in MDI formulations. With diverse size range of SLN, the reverse microemulsion system seemed to exhibit particle confinement capacity (probably endowed by Pluronic L64). All PDI values were lower than 0.25 elaborating a sharp distribution of particle size (Figure 4B). Within 10 d, the average size and PDI did not change significantly (*p* > 0.05), suggesting stable particle size distribution. Moreover, no flocculation, sedimentation, and layering phenomenon were observed during the 10 d storage, strengthening the fact of satisfactory stability.

### 3.3. Safety Evaluation

**Figure 5:**
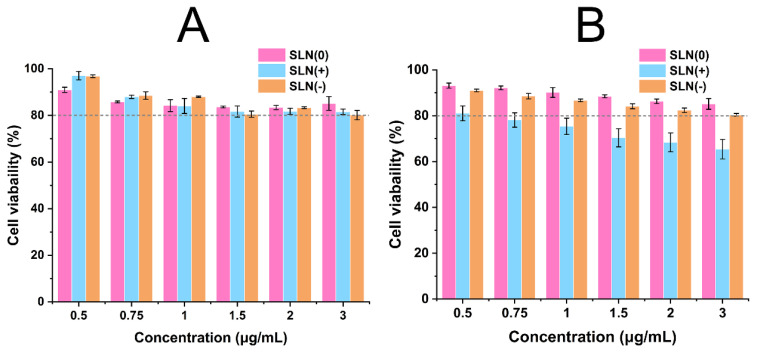
Safety evaluation: cell viability at 24 h (**A**) and 72 h (**B**) of MDI products prepared by SLN(0), SLN(+), and SLN(−) (*n* = 4).

The biocompatibility of MDI products l was assessed through MTT assay. The results are presented in Figure 5. At 24 h (Figure 5A), the viability of A549 cells (human lung carcinoma cell line) was higher than 80% at 0.50~3.00 μg·mL^−1^ concentrations, suggesting good biocompatibility of all the MDI formulations prepared from SLN(0) and SLN(+) and SLN(−). The cell viability of SLN(+) dropped below 80% at 0.75 μg·mL^−1^ or higher concentrations after 72 h, whereas no change was recorded in case of SLN(0) and SLN(−). This phenomenon could be explained by the contribution of inherent cytotoxicity of CTAB [22]. CTAB is absent in SLN(0) whereas, it was veiled by HA layer in SLN(−) and therefore effect was minimized. It could also be conjectured that Poloxamer 188 and HA nanocoating had neglectable cytotoxicity. In summary, the MDI products, SLN(0) and SLN(−) might have acceptable safety in pulmonary delivery. As the volume of lung lining fluid was about 25 mL and given the maximum concentration in this work (3.00 μg·mL^−1^), it could be roughly calculated that SLN(0) and SLN(−) dose up to 75 μg was tolerable after administration.

Above-mentioned results elaborated the feasibility and reproducibility of the proposed production strategy of SLN-based MDI products and suggested that SLN(0) and SLN(−) could be exploited for their industrialization potential.

## 4. Discussion

### 4.1. Study-Design Aspect

The industrialization of inhalable nanoparticles is always a difficult task for the academia and industry. The main problems are: (1) How to obtain nanoparticle with reproducibility on a large scale? (2) How to transform nanoparticle into MDI with reproducibility on a large scale? Answering these two questions stepwise, will help in resolving the industrialization dilemma.

For question (1), since most nanoparticle systems under development were of sophisticated design, it was typically hard to produce those nanoparticles with reproducibility. It was worth noticing that lipid-based nanoparticle was an inspiring exception with great scale-up potential [23]. Therefore, we selected SLN, a representative lipid-based nanoparticle for subsequent study. As shown by the results in Section 3.1, the prepared SLN including SLN(0), SLN(+), and SLN(−), were of reproducible quality and potentially answering the question (1).

The transformation of SLN into MDI is more complicated than the transformation of pure drug into MDI. Physical stability of SLN (mainly mirrored by the particle size [24]) is critical which has no relevance in the case of pure drug. Pluronic L64 was added during the MDI filling process to enhance the physical stability. It could serve as an emulsifier for suspending SLN in the propellant of MDI formulation [17]. Demonstrated in the results Section 3.2, SLN-based MDI with good reproducibility and physical stability could be obtained. As a consequence, question (2) could be addressed.

We must notice that in addition to the formulation design, the credibility of SLN sonication instrument and MDI filling machine played an unneglectable role in addressing questions (1) and (2), respectively. Attention must be paid to the instrument configuration and functions during the development of SLN-based MDI.

The scale-up potential of MDI products of three SLN systems, namely SLN(0), SLN(+), and SLN(−), was demonstrated. The above workflow might also be applicable for the development of other similar systems, most probable for lipid-based nanomedicines, e.g., liposomes, cubosomes, or exosomes. We perceived that this work could act as a precedent for further attempts for inhalable nanoparticle industrialization.

### 4.2. Cytotoxicity Aspect

SLN(0) and SLN(−) showed good biocompatibility in comparison to SLN(+). The reason is the presence of CTAB in SLN(+). Various cationic materials has been reported to be toxic, even including the most commonly used cationic lecithin for cationic liposome preparation, i.e., 1,2-dioleoyl-3-trimethylammonium-propane (DOTAP) [25]. The underlying mechanism was the strong interaction between cationic materials and anionic cell membrane which disturbs the physiological structure and function of the latter [26]. In this work, CTAB was preliminarily chosen because it was involved in the established method by our group [27]; we also expected to accelerate the scale-up of that previously developed inhalable nanoparticle. We perceived that a mere modification of outer layer of SLN with CTAB or similar cationic materials was inappropriate for clinical use. Considering that the CTAB nanocoating was intended for cellular uptake enhancement, we inferred that SLN(+) might be used for the establishment of respiratory injury laboratory models [28] especially after loading with positive inducing agents such as lipopolysaccharides (LPS). We might witness clinical translation of systems such as SLN(0) and SLN(−) in the near future. SLN(0) could be used to deliver a wide range of therapeutic drugs and due to the recognition of HA toward CD44 receptors on tumor cell membrane [29], SLN(−) are prospective in targeted cancer therapy.

Noticeably, a single pilot cytotoxicity examination was insufficient to confer the clinical safety. The cytotoxicity effect on multiple cell lines (Calu-3, Beas-2b, etc.) and safety test on animal models (mouse, rat, dog, etc.) will be reported in due course.

### 4.3. Limitation Aspect

Although this work put forward a strategy to boost the industrial translation of SLN-based MDI by addressing the abovementioned two main questions related to stability of the formulation, there are still some limitations. For instance, SLN carriers without cargoes were employed in the tests. We elucidated the feasibility and reproducibility of SLN (carrier only)-based MDI and planning to examine the scenario of SLN (drugs encapsulated)-based MDI. Based on the A549 (lung cancer cells) model employed in this study, we will first incorporate anticancer drugs such as cisplatin, paclitaxel, and sorafenib.

## 5. Conclusions

In this study, a proof-of-concept of the industrialization of inhalable nanoparticles was conducted. SLN with different nanocoatings, SLN(0), SLN(+), and SLN(−) were prepared and encapsulated into MDI. The MDI products possessed homogeneous particle size distribution and good stability and performed well in evaluations which include appearance, filling reproducibility, valve precision, leak detection, and re-dispersity. SLN(0) and SLN(−) showed good biocompatibility tested against A549 cells (with >80% at XX), while SLN(+) exerted observable cytotoxicity. We suggested to pay more attention to the scale-up for MDI products based on SLN(0) and SLN(−) in the current stage.

## Data Availability

The data presented in this study are available on request from the corresponding author.

## Supplementary Materials

The following supporting information can be downloaded at: https://www.mdpi.com/article/10.3390/pharmaceutics15030866/s1, Figure S1: Evaluations of SLN-based MDI: Photographs of MDI products based on SLN(0) (A), SLN(+) (B) and SLN(−) (C); Table S1: Qualitative solubility test of palmityl palmitate at room temperature.
