# Supplementary material for: A Pilot Study Exploiting the Industrialization Potential of Solid Lipid Nanoparticle-Based Metered-Dose Inhalers
Source: Pharmaceutics. 2023 Mar 7;15(3):866. doi: 10.3390/pharmaceutics15030866 (PMC10052976; doi:10.3390/pharmaceutics15030866)
Supplement: Supplementary file 1 [file pharmaceutics-15-00866-s001.zip › pharmaceutics-2214257-supplementary.pdf]

pharmaceutics

Supplementary Materials

A Pilot Study Exploiting the Industrialization Potential of Solid Lipid Nanoparticle based Metered-Dose Inhalers

Lei Shu[1,†], Wenhua Wang[2,†], Chon-iong Ng [1], Xuejuan Zhang [1,*], Ying Huang [1], Chuanbin Wu [1], Xin Pan [2] and Zhengwei Huang [1,*]

1 College of Pharmacy, Jinan University, Guangzhou 510632, China
2 School of Pharmaceutical Sciences, Sun Yat-sen University, Guangzhou 510006, China
* Correspondence: zhangxj0223@jnu.edu.cn (X.Z.); huangzhengw@jnu.edu.cn (Z.H.); Tel.: +86-020-39943117 (Z.H.)
† These authors contributed equally to this work.

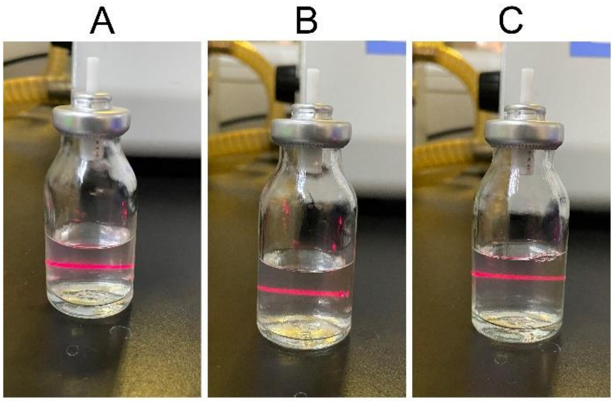

Figure S1. Evaluations of SLN-based MDI: Photographs of MDI products based on SLN(0) (**A**), SLN(+) (**B**) and SLN(-) (**C**).

Table S1. Qualitative solubility test of palmityl palmitate at room temperature.

Solvent	Phenomenon	Roughly-estimated solubility ($mg{\cdot}mL^{-1}$)*
Water	Insoluble	<< 1
Ethanol	Insoluble	<< 1
N,N-Dimethylformamide (DMF)	Insoluble	<< 1
Dimethyl sulfoxide (DMSO)	Insoluble	<< 1
Ethyl acetate	Soluble	> 5
Acetone	Soluble	> 5
Dichloride methane	Easily soluble	>> 10
Trichloride methane	Easily soluble	>> 10

* The value was roughly determined by the following procedures: (1) Add a certain weight of palmityl palmitate into the solvent; (2) Vortex for 1 min and allowed for equilibrium for 24 h; (3) Observe whether the solid was dissolved; (4) Estimate the solubility.
